# *Retraction and Republication:* Timing of Introduction of Complementary Foods — United States, 2016–2018

**DOI:** 10.15585/mmwr.mm7230a6

**Published:** 2023-07-28

On November 27, 2020, *MMWR* published “Timing of Introduction of Complementary Foods — United States, 2016–2018” (*1*), which was based on data from the National Survey of Children’s Health. On April 4, 2023, the National Survey of Children’s Health released a technical document describing a processing error that occurred for data released between 2016 and 2021 (*2*). This processing error caused the reported time in months for breastfeeding, age of first formula, and introduction of solid foods to be incorrectly rounded down by 1 in most cases. Updated data files were released in conjunction with the technical document. *MMWR* was notified about this processing error on May 25, 2023. After discussion with the authors on June 16, *MMWR* published an Expression of Concern on June 30 (*3*), per International Committee of Medical Journal Editors and National Library of Medicine best practices (*4*,*5*).

The authors reanalyzed the updated data files and found that the results and interpretations changed from the original report. Therefore, the original report is retracted. In accordance with December 2017 guidance from the International Committee of Medical Journal Editors (*4*), *MMWR* is republishing the report (*6*). The republished report includes the original, retracted report with clearly marked corrections as supplementary materials.
